# Utilization of Nanotechnology to Improve the Handling, Storage and Biocompatibility of Bioactive Lipids in Food Applications

**DOI:** 10.3390/foods10020365

**Published:** 2021-02-08

**Authors:** David Julian McClements, Bengü Öztürk

**Affiliations:** 1 Department of Food Science, University of Massachusetts Amherst, Amherst, MA 01003, USA; 2 Department of Food Science & Bioengineering, Zhejiang Gongshang University, Hangzhou 310018, China; 3 Department of Food Engineering, Faculty of Engineering, Yeditepe University, Istanbul 34755, Turkey; benguozturk@gmail.com

**Keywords:** nanotechnology, bioactive lipids, storage stability, bioavailability, food applications

## Abstract

Bioactive lipids, such as fat-soluble vitamins, omega-3 fatty acids, conjugated linoleic acids, carotenoids and phytosterols play an important role in boosting human health and wellbeing. These lipophilic substances cannot be synthesized within the human body, and so people must include them in their diet. There is increasing interest in incorporating these bioactive lipids into functional foods designed to produce certain health benefits, such as anti-inflammatory, antioxidant, anticancer and cholesterol-lowering properties. However, many of these lipids have poor compatibility with food matrices and low bioavailability because of their extremely low water solubility. Moreover, they may also chemically degrade during food storage or inside the human gut because they are exposed to certain stressors, such as high temperatures, oxygen, light, moisture, pH, and digestive/metabolic enzymes, which again reduces their bioavailability. Nanotechnology is a promising technology that can be used to overcome many of these limitations. The aim of this review is to highlight different kinds of nanoscale delivery systems that have been designed to encapsulate and protect bioactive lipids, thereby facilitating their handling, stability, food matrix compatibility, and bioavailability. These systems include nanoemulsions, solid lipid nanoparticles (SLNs), nanostructured lipid carriers (NLCs), nanoliposomes, nanogels, and nano-particle stabilized Pickering emulsions.

## 1. Introduction

**Figure 1:**
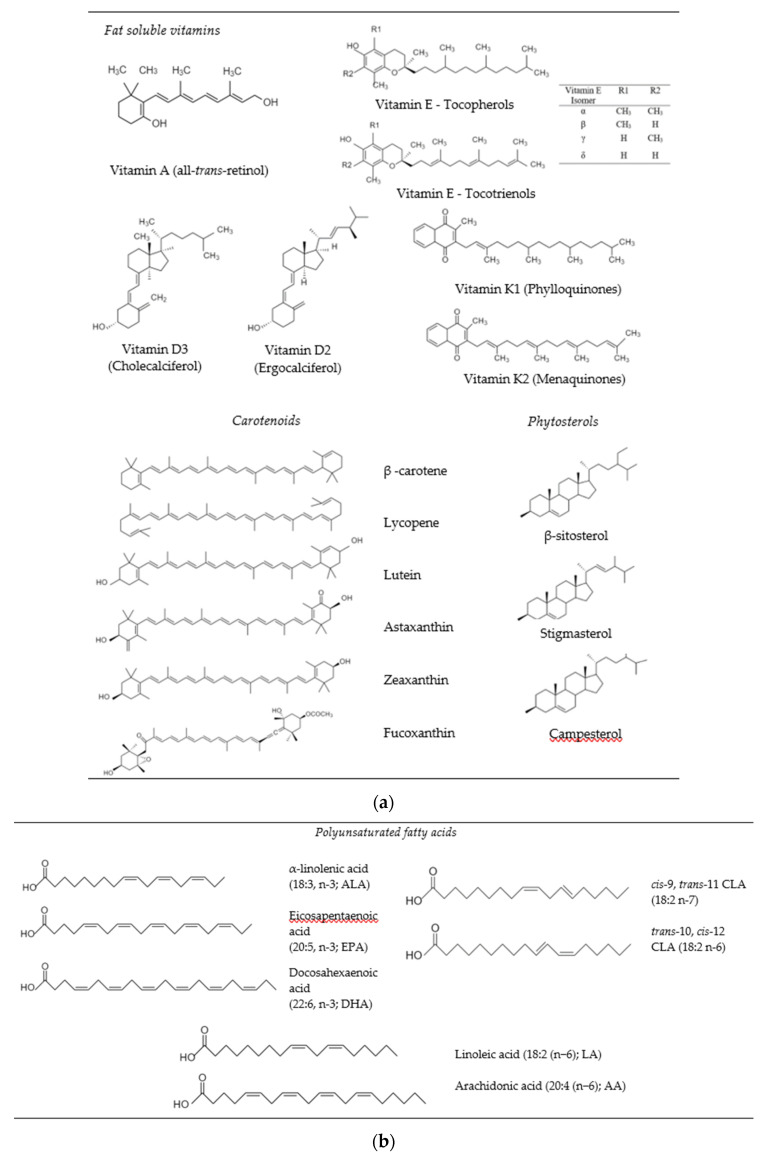
Chemical structures of bioactive lipids: (**a**) fat soluble vitamins, carotenoids, phytosterols, (**b**) polyunsaturated fatty acids.

Bioactive lipids are oil-soluble compounds that may provide certain health benefits to humans when consumed in appropriate amounts, i.e., doses and frequencies. Depending on their mechanisms of action, they may boost the immune system, reduce inflammation, improve bone health, eye and brain functions, reduce coronary heart diseases, and act as antioxidants and anticarcinogens [1,2,3,4]. Some of the most commonly studied bioactive lipids that may promote human health and well-being are phytosterols (e.g., β-sitosterol, stigmasterol, campesterol), fat soluble vitamins (e.g., vitamins A, D, E, K), carotenoids (e.g., β-carotene, lycopene, lutein, zeaxanthin, astaxanthin) and essential fatty acids (e.g., ALA, EPA, DHA, CLA,) [5,6] (Figure 1). As well as their traditional bioactivities (Section 2), a number of studies have recently shown that consumption of some of these bioactive lipids may also provide protection against human coronavirus SARS-CoV-2. For instance, ingestion of EPA and DHA has been reported to reduce inflammation, cytokine storm and blood coagulation, which might play role an important role in the recovery of patients with Covid-19 infection [7,8,9]. Health professionals have also recommended Covid-19 patients to take vitamin D to modulate respiratory tract disorders, boost immune cell proliferation and activity, and promote pulmonary ACE2 expression mechanisms [10].

Most bioactive lipids are not synthesized in the human body and so they have to be obtained from food sources, such as nuts, seeds, grains, eggs, meat, fish, tomato, fruits, and vegetables [1,11,12,13]. However, many people do not get enough of these bioactive compounds from their regular diets due to their low dietary levels, tendency to degrade during processing or storage, and their poor stability, solubility, and absorption characteristics within the human gut [14,15,16,17]. The food industry is therefore exploring methods of fortifying foods with stable and bioavailable forms of these bioactive lipids.

Nanotechnology has emerged as a powerful means of encapsulating, protecting, and delivering bioactive substances in foods, thereby improving their efficacy [18,19]. These nanoscale delivery systems should be constructed entirely from food grade ingredients and should be designed to provide resistance against the elevated temperatures, light levels, and oxygen levels they may be exposed to during food processing and storage. They should also be designed to be stable within the specific food matrix that they are utilized within.

The aim of this review is to highlight the potential of nanoscale delivery systems for improving the handling, storage, and bioavailability of bioactive lipids in foods. Initially, the different kinds of bioactive lipids typically used in foods are introduced, and then the various types of nanoscale delivery systems that can be used to encapsulate them are reviewed. Examples of how these delivery systems are utilized to improve the handling, storage, and bioavailability of bioactive lipids are then given.

## 2. Bioactive Lipids

### 2.1. Fat Soluble Vitamins

Fat soluble vitamins are hydrophobic micronutrients that humans must consume to maintain various body functions and ensure health and wellbeing (Figure 1a) [6,20]. Vitamin A (all-trans retinol) is found in a variety of foods, with the chemical form depending on the source. Retinol is mainly found in plant-based foods while retinyl esters are found in animal-based foods. Vitamin A is known to improve visual health, help reproduction, boost the immune system, and promote growth [21]. Vitamin D is a steroid hormone that comes in two major forms: vitamin D_2_ (ergocalciferol) and D_3_ (cholecalciferol). In the body, the most bioactive form of vitamin D is 1,25-dihydroxyvitamin D_3_, which plays an important role in modulating the immune system, calcium metabolism, and the maintenance of bone and teeth health [22]. Vitamin E is comprised of a number of tocopherols (α-, β-, γ-, and δ-tocopherol) and tocotrienols (α-, β-, γ-, and δ-tocotrienol) that are essential for human health and performance. They also act as natural antioxidants and help prevent cancer, heart disease, diabetes, and skin diseases [23]. Vitamin K is a group of hydrophobic compounds (phylloquinones and menaquinones) that all have a methylated naphthoquinone ring system, which are substituted with isoprenoid and isoprene side chains, respectively. Vitamin K is a micronutrient whose ingestion helps prevent blood clotting and modulates bone health [24].

### 2.2. Carotenoids

Carotenoids are tetraterpenoid lipids that can be divided into two major classes according to their chemical structure: carotenes that are nonoxygenated (e.g., α-carotene, β-carotene, and lycopene) and xanthophylls (e.g., lutein, zeaxanthin, fucoxanthin, and β-cryptoxanthin) that are oxygenated (Figure 1a). Carotenoids are abundant in yellow/orange fruit and vegetables, dark leafy green vegetables, and algae. β-carotene has pro-vitamin A function, since it is converted into vitamin A in the human body through metabolic pathways. Lutein and zeaxanthin retard the progression of age-related cataracts and help to improve cognitive function and cardiovascular health [2,25]. Lycopene has been reported to inhibit breast and prostate cancer, reduced the risk of cardiovascular disease, and retard nerval degenerative diseases [26]. Fucoxanthin can be isolated from seaweed and is claimed to exhibit anticancer, antidiabetic, antioxidant, anti-inflammatory, and antimicrobial activities, was as providing protection against liver, brain, bone, skin, and eye diseases [27,28].

### 2.3. Phytosterols

Phytosterols (plant sterols and stanols) are important structural components of plant cell membranes (Figure 1a) [12]. Sterols can occur in free form or esterified and glycosylated bound form. The most common forms of phytosterols found in foods are β-sitosterol, stigmasterol and campesterol. Phytosterols have been reported to reduce the risk of inflammatory diseases, as well as arteriosclerosis and cardiovascular diseases by lowering serum LDL-cholesterol concentrations [3,29].

### 2.4. Essential Fatty Acids (Omega-3s and CLA)

Alpha-linolenic acid (ALA), eicosapentaenoic acid (EPA), and docosahexaenoic acid (DHA) are the major kinds of omega-3 polyunsaturated fatty acids (PUFAs) found in foods (Figure 1b) [11]. Fish oil is the main source of EPA and DHA in the human diet, although algae oil is also an important source of DHA [30]. Consumption of adequate amounts of omega-3 PUFAs has been reported to reduce the risk of cardiovascular diseases, boost neurological and visual functions, promote healthy aging, and improve cognitive functions in Alzheimer patients [4]. ALA is derived from plant seed oils and can be converted to EPA and DHA in vivo, but at a relatively low conversion rate [31]. Consequently, there is interest in fortifying plant-based foods with ALA as a non-animal source of healthy lipids [32]. Conjugated linoleic acids (CLA) are a group of positional and geometric isomers of linoleic acid. The most common bioactive isomers are *cis*-9, *trans*-11-CLA (9-CLA) and *trans*-10, *cis*-12-CLA (10-CLA), which belong to the n-7 and n-6 family, respectively. In vitro and animal models have shown that CLAs have various potential health benefits, including hypolipidemic, anti-carcinogenic, ostheosynthetic, anti-diabetagenic, and immunomodulatory effects. The major dietary sources of CLA are dairy and meat products from ruminants [33].

## 3. Nanoscale Delivery Systems for Encapsulation of Bioactive Lipids

**Figure 2:**
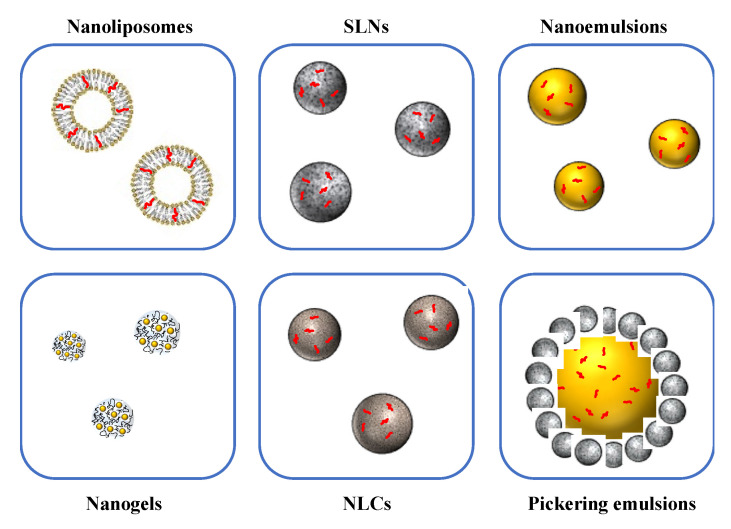
Various kinds of edible nanoparticles are available to encapsulate, protect, and delivery bioactive substances in foods.

**Figure 3:**
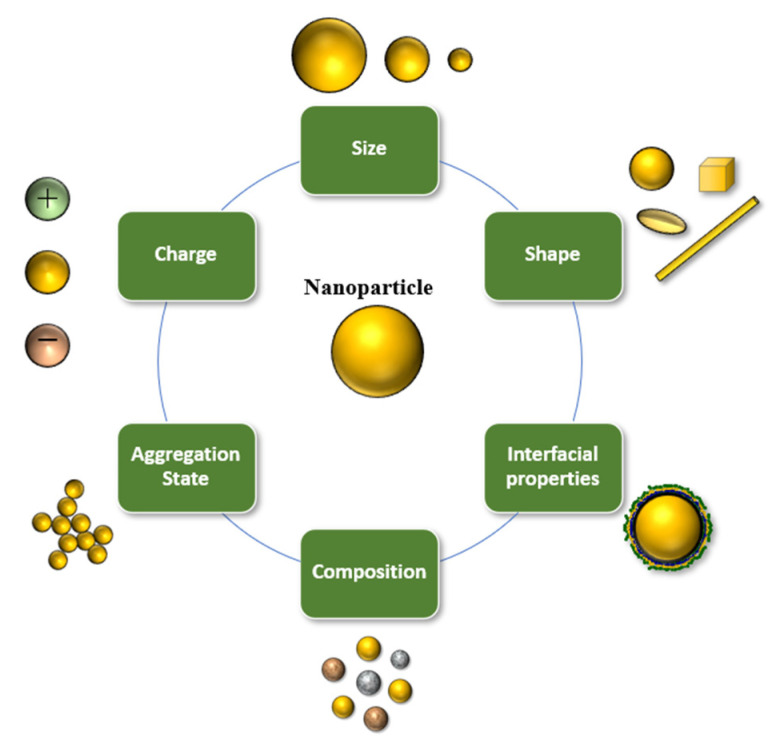
The properties of edible nanoscale delivery systems can be controlled to tailor their functionality.

Nanoscale delivery systems can be used to improve the handling, stability, and bioavailability of bioactive lipids [16,19,34,35]. For specific applications, nanotechnology has advantages over conventional encapsulation technologies, due to the smaller particle size and greater surface area of the carrier particles employed. Several lipid-based nanoscale delivery systems have been developed and to protect and deliver bioactive lipids, which differ in their compositions, structures, and functionalities (Figure 2) [16,36]. The functional performance of many of these delivery systems can be tailored for specific applications by changing the size, shape, charge, composition, or aggregation state of the nanoparticles they contain (Figure 3). A number of the most widely used nanoscale delivery systems for bioactive lipids are highlighted here.

### 3.1. Nanoemulsions

Oil-in-water nanoemulsions consist of small emulsifier-coated oil droplets (*d* < 200 nm) dispersed in water [37] (Figure 2). The bioactive lipids are typically trapped inside the oil droplets, which can be achieved either before or after nanoemulsion formation. This kind of nanoemulsion may be fabricated from food-grade oil, water, and emulsifier using low- or high-energy approaches. Low-energy approaches rely on the spontaneous formation of small oil droplets when the system composition or environmental conditions are changed in a specific fashion. High-energy approaches utilize mechanical devices (“homogenizers”) to break up the oil and water phases and form small oil droplets that are coated by emulsifiers. Numerous food grade emulsifiers can be used to create edible nanoemulsions, including synthetic surfactants, biosurfactants, phospholipids, proteins, and polysaccharides [38,39,40,41]. Typically, the emulsifier-coated oil droplets are prevented from aggregating (flocculating or coalescing) with each other by generating strong electrostatic and/or steric repulsive forces between them. The small size of the droplets in nanoemulsions provides strong resistance to gravitational separation [37]. However, nanoemulsions are susceptible to droplet growth through Ostwald ripening when the oil phase used has an appreciable solubility in water (such as for essential or flavor oils). In this case, they have to be carefully designed to reduce this form of instability, e.g., by adding a ripening inhibitor.

Nanoemulsions should be clearly distinguished from microemulsions, which are also used as bioactive delivery systems and have some similar compositional and structural characteristics [37]. However, nanoemulsions are thermodynamically unstable systems, whereas microemulsions are thermodynamically stable ones. As a result, microemulsions can often be formed by simply mixing bioactive, surfactant, and water together, whereas nanoemulsions require specific preparation methods (often involving high energy input). One drawback of microemulsions is that they can usually only be formulated from high concentrations of synthetic surfactants, which is undesirable for many food applications [42].

### 3.2. Solid Lipid Nanoparticles and Nanostructured Lipid Carriers

Solid lipid nanoparticles (SLN) also contain small emulsifier-coated lipid particles (*d* < 200 nm) but in this case the molecules within the lipid phase are organized into highly regular crystalline structures, rather than being disorganized as in a fluid [43] (Figure 2). The bioactive lipids are again located inside the lipid phase. If they are successfully trapped within the crystal structure formed by the lipid molecules, then they may have improved resistance to chemical degradation and a sustained release rate. However, they are sometimes expelled to the surfaces of the SLNs when the lipid phase crystallizes, which can actually reduce their chemical stability. Moreover, the lipid nanoparticles may change shape from spherical to non-spherical after formation, which can increase their surface area susceptibility to aggregation. For these reasons, the nature of both the lipid phase and emulsifier used must be carefully selected to ensure good performance of SLNs. Nanostructured lipid carriers (NLCs) were designed to overcome the problems with SLNs (Figure 2). They again consist of small emulsifier-coated lipid particles (*d* < 200 nm) dispersed in water, but in this case a mixture of solid and liquid lipids is used to create a less ordered lipid phase, which allows higher solubilization and retention of the bioactive lipids, as well as decreasing changes in particle shape during lipid phase crystallization [44,45].

### 3.3. Nanoliposomes

Nanoliposomes are colloidal dispersions containing small (*d* < 200 nm) lipid nanoparticles comprised of one or more concentric lipid bilayers formed by self-association of phospholipid molecules in water [46] (Figure 2). At the particle surfaces, the polar head groups of the phospholipids protrude into the surrounding water phase. Within the concentric bilayers, the non-polar phospholipid tails face each other, which allows bioactive lipids to be trapped within these hydrophobic domains [34,35,47]. Nanoliposomes may be formed by various methods, including coating-solvent evaporation and homogenization methods [34], with the former being mainly used in the laboratory and the latter being more suitable for large-scale continuous commercial production.

### 3.4. Biopolymer Nanogels and Nanofibers

Biopolymer nanogels and nanofibers have been developed to encapsulate bioactive lipids [35] (Figure 2). Nanogels consist of small biopolymer-rich particles (<200 nm) suspended in water. The particles contain a 3D network of crosslinked biopolymer molecules that trap a considerable amount of water through hydration and capillary forces. The biopolymer molecules may be crosslinked by physical or chemical interactions and the pore size of the network formed can be controlled by altering biopolymer or crosslinking concentration, as well as preparation conditions. Bioactive lipids may be directly encapsulated within the biopolymer network, or they may first be loaded into lipophilic nanocarriers (such as nanoemulsions or nanoliposomes). Biopolymer nanogels can be designed to improve the stability and delivery of bioactive lipids [19,35]. Biopolymer nanofibers, which consist of thin fibers of crosslinked biopolymer molecules, are typically formed using electrospinning methods. Bioactive lipids can be directly or indirectly trapped within the biopolymer solution used to form the nanofibers prior to the electrospinning process. Nanochitin, nanocellulose, and nano-starch delivery systems have been produced using this method for food processing and packaging applications [48,49].

### 3.5. Nanoparticle-Stabilized Pickering Emulsions

Oil-in-water Pickering emulsions consist of oil droplets coated by colloidal particles [50,51] (Figure 2). These types of structured emulsions can be produced entirely from food grade ingredients and can be used to encapsulate bioactive lipids. Food grade nanoparticles that are suitable for used as emulsifiers in Pickering emulsions include nanocellulose, nanochitin, nano-starch, protein nanoparticles, and SLNs [51,52,53,54]. The bioactive lipids can be encapsulated within the oil droplets or within the nanoparticles used to stabilize them.

### 3.6. Nanoparticle Formation Methods

Nanoscale delivery systems can be produced using various low- or high-energy approaches, depending on the nature of the bioactive to be encapsulated, the nature of the encapsulating materials used to assemble the particles, and the intended functional attributes of the final system. High-energy methods include microfluidization, sonication, homogenization, and high-speed shearing methods, while low-energy methods include spontaneous emulsification, phase inversion, antisolvent precipitation, and evaporation methods [16,34,44]. Nanoemulsions, SLNs, and NLCs are commonly produced using both high-energy (sonication, homogenization, microfluidizer) and low-energy (spontaneous emulsification, phase inversion) methods. In the case of SLNs and NLCs, a nanoemulsion is typically prepared at high temperatures, which is then cooled below the melting point of the lipid phase [43]. Nanoliposomes can also be produced using both low- and high-energy methods, such as coating-evaporation and microfluidization approaches [16]. Other technologies, such as nano-spray drying and freeze drying can be used to create powders containing nanoparticles loaded with bioactive lipids [55,56,57,58,59]. Each nanoscale delivery system has its own pros and cons, which should be considered when selecting one for a specific food application.

## 4. Improved Food Matrix Compatibility and Handling

Encapsulation of bioactive lipids in nanoparticles is widely used to improve their compatibility with different kinds of food matrices. The exterior of these nanoparticles is usually hydrophilic, which means they can be readily dispersed into the aqueous environments found in many foods and beverages, such as soft drinks, milk analogs, yogurts, dressings, sauces, and condiments. Nevertheless, the nanoparticles must also be designed to remain stable under the specific solution conditions found within a particular food product, such as its pH, ionic strength, and ingredient formulation. This means that any repulsive interactions between the nanoparticles, typically electrostatic and/or steric, must be designed to be stronger than any attractive interactions, typically van der Waals, hydrophobic, bridging and/or depletion [60]. This can be achieved by controlling the interfacial composition and structure, particularly the charge, thickness, and polarity of the nanoparticle coating.

Nanotechnology can also be used to improve the handling and storage of bioactive lipids. For instance, the bioactive lipids can be converted into a liquid, gel, powdered, or solid form that can facilitate their transport and utilization during the manufacturing process. Moreover, trapping the bioactive lipids within nanoparticles can improve their resistance to creaming, sedimentation, flocculation, or coalescence during storage, as well as their resistance to chemical degradation, thereby increasing the shelf-life of the food product [37]. Nevertheless, nanoscale delivery systems must be carefully designed to retain the encapsulated bioactive lipids throughout storage, as well as to protect them from the specific environmental stressors they are exposed to, such as pH changes, heating, cooling, shearing, and dehydration. In the remainder of this section, examples of some of the nanoparticle-based systems used to improve the food matrix compatibility or handling of bioactive lipids are given.

Nanoliposomes have been used to incorporate fish oil into yogurts so as to fortify them with health-promoting omega-3 fatty acids [61]. Nanoencapsulation of the fish oils significantly increased the overall acceptance and taste, aroma, texture, and appearance of the yogurts compared utilization of non-encapsulated (free) fish oil. Fish oil has also been encapsulated within caseinate-gum arabic nanocomplexes that were converted into a powdered form and then introduced into fruit juices [62]. Sensory analysis of these omega-3 enriched fruit juices (60 mg EPA+DHA/100 mL) indicated an acceptable taste by consumer panelists, suggesting that nanoencapsulation masked off-flavors from the fish oil. In another study, fish oil was encapsulated within nanoliposomes that were then incorporated into bread, without adversely affecting the texture or sensory acceptability [63]. Conjugated linoleic acid (CLA) has been encapsulated within NLCs that were then added to low-fat milk [64]. Encapsulation was shown to improve the oxidative stability of the CLA, thereby extending the shelf life of the product.

Nanoencapsulation has also been used to improve the food matrix compatibility of various kinds of carotenoids. For instance, astaxanthin has been encapsulated within oil-in-water nanoemulsions that were then successfully introduced into orange juice and milk to form stable carotenoid-fortified products [65]. Zeaxanthin has been encapsulated in biopolymer nanoparticles and nanoemulsions and then incorporated into yogurt [66]. Encapsulation of the carotenoids improved their chemical stability, while having no adverse effects on the appearance, color, flavor, consistency, and texture of the fortified yogurts. Carotenoids extracted from Cantaloupe melon have also been encapsulated in nanoemulsions stabilized by whey proteins and gelatin, which were then incorporated into yogurt [67]. In this case, nanoencapsulation improve the dispersibility and color stability of the carotenoids in the yogurt compared to a crude carotenoid extract. Lycopene has been encapsulated within SLN and NLC systems, which were then successfully introduced into an orange drink without adversely affecting its sensory attributes [68].

Powdered forms of bioactive-loaded nanoparticles can be produced by spray drying, which improves the handling and storage stability. The powders produced can be directly incorporated into dried food products, such as soup mixes, fruit powders, cereal powders, and infant formulas, by simple mixing, or they can be redispersed in water and then incorporated into wet food products, such as beverages, soups, or sauces [69]. Flaxseed oil (56% α-linolenic acid) has been encapsulated within nanocomplexes formulated from high amylose corn starch, which were then converted into a powdered form using spray drying [57]. The resulting powder was then used to fortify bread with omega-3 fatty acids. The encapsulated flaxseed oil had higher oxidative stability than free flaxseed oil during baking, which reduced off-flavor and acrylamide formation.

Vitamin D_3_ has been encapsulated within NLCs, which were then introduced into a yogurt-based beverage (“Lassi”), without adversely affecting the sensory attributes of the fortified product, such as appearance, flavor, aftertaste, and homogeneity [70]. Plant-based nanoemulsions have been used to encapsulate vitamin D and introduce them into plant-based milk analogs, such as almond and oat milks [71]. The nanoemulsions did not negatively alter the color or viscosity of the fortified milk analogs. Some examples of food and beverages that have been fortified with nanoencapsulated bioactive lipids are provided in Table 1.

## 5. Enhanced Stability

It is important that bioactive lipids remain stable after being incorporated into delivery systems or into fortified foods. A number of bioactive lipids are chemically unstable and so may degrade during the processing or storage of fortified foods, i.e., omega-3 fatty acids, CLA, vitamin E, and carotenoids [17]. These instability reactions are often accelerated by exposure to heat, oxygen, light, or pro-oxidants. In some cases, the chemical instability of bioactive lipids reduces their biological efficacy. In other cases, labile bioactive lipids can be used as natural antioxidants to prevent the oxidation of other labile bioactive lipids, e.g., carotenoids can be used as antioxidants for omega-3 fatty acids. As well as their chemical instability, it is also important to inhibit the physical instability of bioactive lipids within foods during processing, storage, and utilization, e.g., due to aggregation or gravitational separation.

Nanoencapsulation can be used to overcome problems associated with the chemical and physical stability of bioactive lipids. Vitamin A has been encapsulated within SLNs and NLCs using hot homogenization, which was shown to produce a physically and chemically stable form of these bioactive lipids [78]. Vitamin D_3_ has been encapsulated within protein-polysaccharide nanocomplexes [79], which were shown to remain physically and chemically stable for 31 days when stored at 4 °C. In another study, it was shown that encapsulation of vitamin A in caseinate nanocomplexes improved its storage stability compared to free vitamin A [80]. The stability of vitamin D_2_ in milk to pasteurization, boiling, and sterilization improved after it was encapsulated within caseinate nanocomplexes [74]. Moreover, the stability of the encapsulated vitamin D_2_ was higher than that of free vitamin D_2_ during 6-months storage of the fortified milks at −20, 4, and 37 °C, as well as when the milks were exposed to light. Nanoemulsions fortified with vitamin D_3_ were shown to remain relatively stable when exposed to a range of environmental stresses, including heating, chilling, pH variations (pH 3 to 7), and ionic strength (0–750 mM) [81]. The highest retention was achieved under refrigerated conditions, at lower ionic strengths, and at pH 7.0. Encapsulation within nanoemulsions has also been shown to improve the oxidative stability of vitamin E during long-term storage [82]. Vitamin E encapsulated within nanoemulsions has been used to fortify mango juice [75], leading to a product that had good physical and chemical stability during storage. Nanoemulsions have also been shown to improve the stability of vitamin E to thermal degradation when exposed to various heat treatments, i.e., 30–90 °C for 30 min [83]. Rapid degradation of vitamin E was only observed at the highest temperature used (90 °C). The vitamin E nanoemulsions exhibited good long-term stability (60 days) when stored in the light and in the dark at 4 and 25 °C, but some degradation was observed during storage at 40 °C. The authors also showed that the storage stability of the vitamin E was higher when long-chain triglycerides were used as an oil phase rather than shorter chain ones. Nanoemulsions have also been shown to improve the chemical stability of vitamin E during 4-weeks storage in the dark [84]. Reducing the oil droplet size in these nanoemulsions also improved the storage stability of the nanoemulsions to gravitational separation.

The stability of β-carotene encapsulated within nanoemulsions has been examined when they were exposed to thermal treatments (pasteurization and sterilization), as well as they were stored in the light and the dark at 4, 25, and 37 °C for up to 90 days [85]. Storage at 4 °C in the dark was found to be the best conditions to prevent β-carotene degradation.

Encapsulation of lutein within nanoemulsions stabilized by food grade emulsifiers has been shown to improve the chemical stability of the carotenoid in nutraceutical-fortified beverages [86]. The physical stability of the nanoemulsions to aggregation and creaming could be controlled by selecting an appropriate emulsifier. The physical and chemical stability of lycopene encapsulated in nanoemulsions has been determined when they were stored at 4, 20, 30 and 40 °C for 3 weeks in the dark [72]. The results showed that the rate of lycopene degradation decreased as the temperature decreased.

DHA has been successfully encapsulated within casein micelles (r-CM) and casein nanoparticles (CNP), which improved the chemical stability of the polyunsaturated lipids during storage at 4 °C for 16 and 37 days [87]. DHA has also been encapsulated within β-lactoglobulin-pectin nanodispersions, which improved its resistance to oxidation when exposed to an accelerated shelf-life stress test, i.e., 100 h at 40 °C [88]. Indeed, there was only 5–10% degradation of DHA in the encapsulated form compared to 80% for the bulk form. The encapsulation of krill oil, which is high in omega-3 fatty acids, within nanoemulsions upon the stability of the oil to oxidation during freeze drying, spray drying, and storage for 12 days has been examined [58]. Encapsulation in the nanoemulsions actually reduced the oxidative stability of the krill oil, which was attributed to the increased exposure of the oil phase to air as a result of the increase in surface area. The addition of a natural antioxidant (vitamin E) has been shown to improve the oxidative stability of ALA encapsulated within nanoemulsions [89]. In this case, the encapsulated ALA had a higher oxidative stability than the free form. Higher temperatures and the presence of metal ions increased ALA degradation, whereas light exposure and ionic strength did not significantly change ALA degradation.

The physical and chemical stability of omega-3, β-carotene and vitamin A encapsulated within SLNs has been studied during 51-days storage [17]. The physical stability of the bioactive-loaded SLNs could be improved by using suitable surfactants, such as *quillaja saponin* and its combination with high-melting lecithin. The oxidative stability of the encapsulated bioactive lipids was improved by preventing the polymorphic transition of the lipid phase in the SLNs by using high-melting surfactants. The oxidative stability of EPA and DHA was improved after encapsulation within nanoliposomes [90], while that of ALA and quercetin was improved after co-encapsulation in NLCs [91]. Encapsulation of bixin (a carotenoid) within nanocapsules was found to improve its chemical stability when exposed to light, oxygen, and heat [92]. A phytosterol (β-sitosterol) has been encapsulated within NLCs, which were then incorporated into butter [76]. Fortification of the butter with β-sitosterol increased its resistance to oxidation during long-term storage (3-months) in a refrigerator.

Pickering emulsions stabilized with food-grade nanoparticles have been reported to be good candidates for use in processed foods due to their higher stability against environmental stresses such as freeze-thawing, extreme pH conditions, and high ionic strengths [50]. Pickering emulsions stabilized by chitosan hydrochloride/carboxymethyl starch nanogels have been used to encapsulate β-carotene, which improved the stability of the carotenoid to UV exposure and heating [93]. Pickering emulsions stabilized by egg yolk peptides and micellar nanoparticles were found to be stable to spray drying conditions and to oil leakage during storage [54]. Similarly, encapsulation of β-carotene in Pickering emulsions stabilized by gliadin nanoparticles and gum arabic has been shown to improve the resistance of the carotenoid to pH, salt, heat, and light [94]. Pickering emulsions have also been used to improve the chemical stability of astaxanthin [95]. In this case, the Pickering emulsions were stabilized by lupin protein aggregates.

## 6. Enhanced Bioavailability

**Figure 4:**
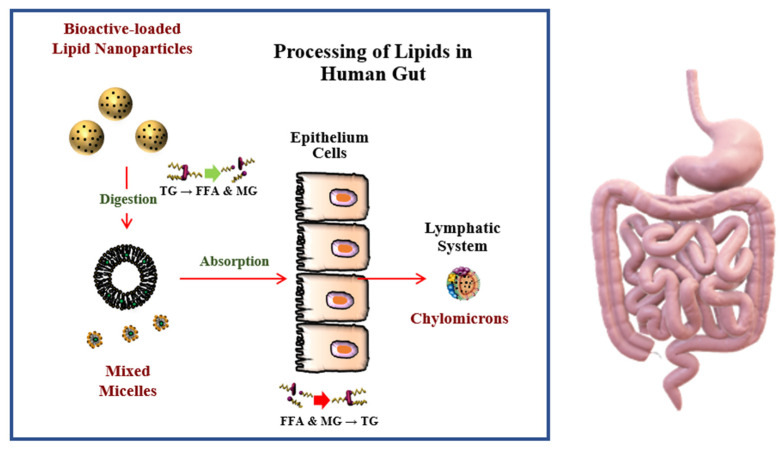
The bioavailability of hydrophobic bioactive substances can be increased by encapsulating them in nanoparticles. The triglycerides (TG) in the lipid nanoparticles are broken down to free fatty acids (FFA) and monoglycerides (MG), which are packed into mixed micelles with bioactive substances and transported to the epithelium cells. They are then reassembled into triglycerides, packed into chylomicrons, and transported into the bloodstream through the lymphatic system. Lipid digestion and nutraceutical bioaccessibility increases with decreasing droplet size.

The bioavailability of bioactive substances is influenced by their liberation, solubilization, absorption, and metabolism within the gastrointestinal tract (GIT) (Figure 4) [96]. For lipophilic bioactive substances, the rate limiting steps determining their bioavailability is often their poor solubilization within intestinal fluids, as well as their chemical degradation. The bioavailability of these lipophilic bioactives can therefore be improved by increasing their bioaccessibility and inhibiting their degradation within the gastrointestinal tract [96]. Nanoencapsulation has been widely used to improve the bioavailability of this type of bioactive substance [18].

Lipid nanoparticles undergo a number of disassembly and assembly processes inside the human body that impacts the bioaccessibility of any hydrophobic bioactives encapsulated within them [97]. The nanoparticles are digested by lipases in the stomach and small intestine, which releases free fatty acids and monoacylglycerols that are incorporated into mixed micelles with bile salts and phospholipids. These mixed micelles then solubilize the hydrophobic bioactives and transport them to the epithelial cells where they can be absorbed. After absorption, the hydrophobic bioactives are packed into chylomicrons that carry them into the bloodstream via the lymphatic system, thereby by-passing first-pass metabolism within the liver [98]. The majority of bioavailability studies have been carried out using in vitro simulated gastrointestinal tract (GIT) models due to their relatively low cost, simplicity, and speed. However, researchers may also use Caco-2 cell lines or animal models to study the bioavailability of bioactive lipids. In particular, co-cultured cell lines containing different kinds of cells (such as model enterocytes, M-cells, and mucus-secreting cells) are particularly useful for mimicking the lining of the human GIT [99,100,101].

As an example, omega-3 fatty acids loaded into nanoemulsions have been shown to increase the bioaccessibility of EPA and DHA compared to bulk oil using a simulated GIT model [102]. Encapsulation of astaxanthin in potato protein-based nanoparticles was shown to improve the bioaccessibility, chemical stability, and bioaccessibility of the carotenoids under simulated gastrointestinal conditions [103]. For instance, encapsulation increased the bioaccessibility by 11-fold and the bioavailability by 7-fold.

The encapsulation of vitamin A into caseinate nano-complexes has been shown to increase the in vitro bioaccessibility and bioavailability of this fat soluble vitamin [104]. In particular, nanoencapsulation protected the vitamin A from degradation under the acidic conditions in the simulated stomach, as well as increasing the fraction absorbed by Caco-2 cells compared to the free form. NLCs have been used to increase the bioavailability of vitamin D_3_, which again was partly attributed to its ability to increase the chemical stability under simulated gastric conditions [105]. Similarly, encapsulation of vitamin D_3_ in ovalbumin-pectin nanocomplexes has been shown to protect the vitamin from degradation under gastric conditions [79].

Oil-in-water nanoemulsions stabilized by a natural surfactant (quillaja saponin) have been shown to increase the bioaccessibility of vitamin D_3_ [106]. The nature of the oil phase has been shown to have a major impact on the bioaccessibility of lipophilic bioactives. For instance, vitamin E encapsulated within nanoemulsions formulated from long-chain triglycerides (LCT) had a higher bioaccessibility than those formulated from medium-chain triglycerides (MCT) [107]. In another study, encapsulation of gamma- and delta-tocotrienols in nanoemulsions was shown to increase their bioaccessibility compared to conventional emulsions using a simulated GIT [108]. In addition, encapsulation of vitamin E in plant-based nanoemulsions was shown to increase its bioaccessibility by an amount that depends on oil phase composition [84].

Encapsulation of lutein within sophorolipid-coated zein nanoparticles has been shown to improve the bioaccessibility of the carotenoid using a GIT model [109]. Similarly, encapsulation of lycopene into oil-in-water nanoemulsions increased carotenoid bioaccessibility, with the efficacy increasing with decreasing droplet size [110]. Phytosterols have been encapsulated within pectin-coated zein nanoparticles, which was shown to improve their stability and to modulate their release under simulated GIT conditions [111]. Phytosterols have also been encapsulated within nanoporous starch aerogels [77], which increased their in vitro bioaccessibility when incorporated into granola bars and puddings compared to free phytosterols.

Excipient foods are designed to increase the bioavailability of bioactive substances in other foods that are co-ingested with them. Excipient nanoemulsions have been used to enhance the oral bioavailability of various kinds of bioactive lipids, which has been attributed to their ability to increase the bioaccessibility, retard the chemical degradation, and increase the absorption of these compounds [112]. For instance, oil-in-water nanoemulsions have been used to increase the bioaccessibility of lycopene in tomato juice [113]. This effect was attributed to the rapid formation of mixed micelles in the small intestine, which could solubilize the lycopene. Nanoemulsions have also been used to increase the in vitro bioaccessibility and absorption of β-carotene using simulated GIT models, which was again attributed to increased mixed micelle formation [93,114]. Encapsulation of EPA and DHA within nanoemulsions has also been shown to increase their in vivo bioavailability using rat models [115]. In another study, the oral bioavailability of vitamin E (γ-tocotrienol) was enhanced three-fold when orally administered to rats in the form of SLNs [116]. Overall, all of these studies show that various kinds of edible nanoemulsions can be used to increase the bioavailability of bioactive lipids.

## 7. Conclusions

There are a wide variety of different bioactive lipids found in foods that may exhibit health benefits in humans. However, these benefits may not be fully realized because many bioactive lipids are difficult to incorporate into foods, are degraded in foods during storage, or have low bioavailability after ingestion. Nanotechnology can be used to improve the matrix compatibility, physiochemical stability, and bioavailability of bioactive lipids. In particular, edible nanoparticles can be created from food grade ingredients to overcome these challenges in food applications. This review has highlighted the characteristics of different kinds of bioactive lipids (e.g., oil-soluble vitamins, carotenoids, phytosterols, and essential fatty acids), as well as the different types of nanoscale delivery systems that can be used to encapsulate them (such as nanoemulsions, SLNs, nanoliposomes, biopolymer nanogels, and Pickering emulsions). In the future, it will be important to identify the most appropriate nanoscale delivery systems for each application, to test the optimized delivery systems using in vivo animal and human studies, and to ensure that the delivery systems remain viable within real food applications.

## Figures and Tables

**Table 1:** Effect of nanoencapsulation on applications of bioactive lipids in foods and beverages.

Bioactive Lipid	Nanocarrier System	Food Application	Benefits	Reference
*Polyunsaturated omega-3 and omega-6 fatty acids*				
Flax seed oil (high in ALA)	Starch nanocomplexes	Bread	Reduced lipid oxidation, reduced HMF and acrylamide during baking	[57]
Fish oil (EPA-DHA)	Nanoliposomes	Yogurt	Increased bioactive retention, reduced lipid oxidation, high sensory score	[61]
Fish oil (EPA-DHA)	Sodium caseinate/gum arabic nanocomplexes	Fruit juice	Acceptable taste scores, high bioaccessibility	[62]
Fish oil (EPA-DHA)	Nanoliposomes	Bread	Improved oxidative stability, good textural and sensory quality	[63]
CLA	Nanostructured lipid carriers	Low fat milk	Improved physical, oxidative and thermal stability	[64]
*Carotenoids*				
Astaxanthin	Nanodispersions	Orange juice, skimmed milk	Improved storage stability, high in vitro cellular uptake	[65]
Zeaxanthin	Nanoparticles, nanoemulsions	Yogurt	High storage stability in food matrix, in vitro controlled release	[66]
Carotenoid extract (high in β-carotene)	Nanoemulsion	Yogurt	Increased dispersibility, high storage stability	[67]
Lycopene	Solid lipid nanoparticles, nanostructured lipid carriers	Orange drink	Increased dispersibility, better aftertaste scores, high overall acceptance	[68]
Lycopene	Nanoemulsion	Model beverage	Improved storage stability	[72]
*Fat soluble vitamins*				
Vitamin D_3_	Nanostructured lipid carrier	‘Lassi’ Yogurt based beverage	High sensory acceptance, sustained release in simulated intestinal fluid	[70]
Vitamin D_3_	Organic nanoparticles (Nanoemulsion, Nanocellulose), inorganic nanoparticle (TiO_2_)	Plant-based milks	Improved viscosity and color	[71]
Vitamin A, D_3_	Reassembled casein micelle	Skim milk	Improved storage stability	[73]
Vitamin D_2_	Na-caseinate nanocomplexes	Milk	Improved storage and thermal stability	[74]
Vitamin E	Nanoemulsion	Fruit juice	Increased shelf life	[75]
*Phytosterols*				
β-sitosterol	Nanostructured lipid carriers	Butter	Improved storage stability, high antioxidant activity	[76]
Phytosterols	Nanoporous starch aerogels	Granola bars, pudding	Improved bioaccessibility	[77]

ALA: α-linolenic acid, EPA: eicosapentaenoic acid, DHA: docosahexaenoic acid, CLA: conjugated linoleic acid.
